# Resolving the Architecture and Early Evolution of a Forearc Basin (Georgia Basin, Canada) Using Detrital Zircon

**DOI:** 10.1038/s41598-019-51795-5

**Published:** 2019-10-25

**Authors:** Chuqiao Huang, Shahin E. Dashtgard, Bryan A. P. Kent, H. Daniel Gibson, William A. Matthews

**Affiliations:** 1 Applied Research in Ichnology and Sedimentology (ARISE) Group, Department of Earth Sciences, Simon Fraser University, Burnaby, B.C. V5A 1S6 Canada; 2 Department of Geoscience, University of Calgary, Calgary, AB T2N 1N4 Canada

**Keywords:** Stratigraphy, Sedimentology

## Abstract

Convergent-margin basins (CMBs) are commonly associated with active arcs, and hence are rich in detrital zircon (DZ) whose ages closely reflect the timing of deposition. Consequently, maximum depositional ages (MDA) from DZ geochronology can be employed to resolve the stratigraphy and evolution of CMBs. Herein, we use DZ to revise the internal architecture of the lower Nanaimo Group, which partially comprises the fill of the (forearc) Georgia (or Nanaimo) Basin. Maximum depositional ages and multi-dimensional scaling of DZ age distributions are employed to determine chronologic equivalency of strata and assess sediment provenance variability within the pre-existing lithostratigraphic framework. The results are compared to a recently developed sequence stratigraphic framework for the lower Nanaimo Group. The basal lithostratigraphic unit of the Nanaimo Group, the Comox Formation (Fm), comprises strata that are neither time correlative nor genetically related. The three lithostratigraphic units directly overlying the Comox Fm (Haslam, Extension, and Protection formations) comprise strata with similar genetic affinities and MDAs that indicate deposition of these units was not always sequential and locally was contemporaneous. Through this work, we provide an example of how MDAs from DZ geochronology in CMBs can resolve basin-scale stratigraphic relations, and identify chronological changes in sediment provenance.

## Introduction

**Figure 1:**
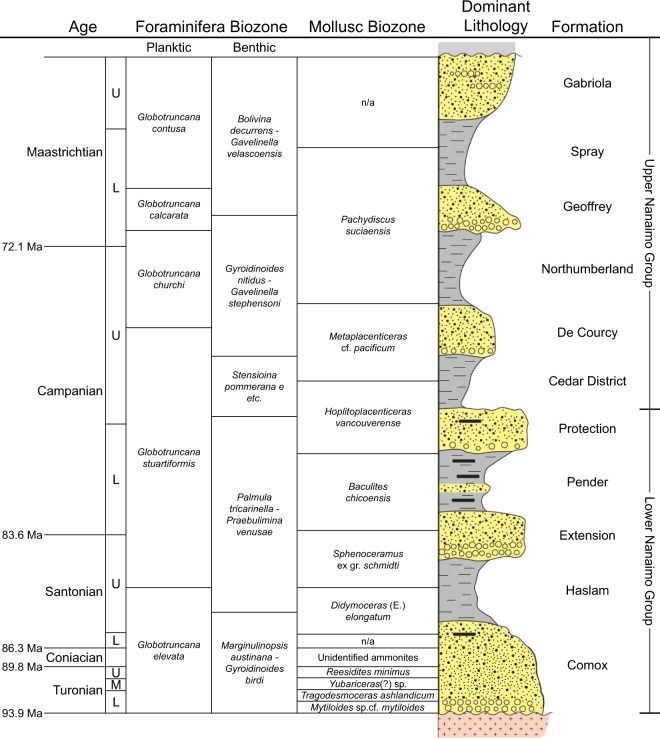
Nanaimo Group lithostratigraphy^1^, Georgia Basin, Canada including biozones as defined by biostratigraphy^21,23,24^. In the Dominant Lithology column, gray indicates mudstone/shale, yellow indicates sandstone and conglomerate, and black indicates coal. U = upper, M = middle, L = lower. Mbr = member. Figure modified after Mustard^1^, with data from England^26^ and Hamblin^22^.

Lithostratigraphy organizes strata based on lithology, and was not intended to determine time-equivalency of strata or reconstruct basin evolution. Despite this, the strata of many convergent-margin basins (CMBs), including forearc basins such as the Georgia Basin (also known as the ‘Nanaimo Basin’), Canada, remain characterized mainly by lithostratigraphic and/or biostratigraphic frameworks (Fig. 1)^1–4^, leading to continuing uncertainties regarding the architecture of the basin fill and its depositional history. Although sequence stratigraphic frameworks can be used to resolve chronological and spatial relations of strata, the long-distance stratigraphic correlations necessary for sequence stratigraphic analysis are challenging to establish in CMBs as these basins commonly experience extensive structural modification and possess multiple disconformities.

Convergent margin basins typically form in close proximity to active arcs, and their fill is rich in contemporaneous detrital zircon (DZ) as a result^5^. The abundance of such DZ can be used to refine depositional ages and sediment provenance. Specifically, maximum depositional ages (MDAs) from DZ calculated using single youngest grain or youngest grain cluster techniques have been shown to approximate true depositional age (TDA) in many CMBs^5–8^ and can be used as a “first pass” for subdividing stratigraphy and informing long-distance stratigraphic correlations^9–11^. In the Georgia Basin, Canada, magmatism in the nearby Coast Plutonic Complex (CPC), a long-lived continental arc^12–14^, provided significant detritus for the Nanaimo Group that included DZ broadly coeval with deposition^1,7,12,15–18^. Herein we present high-n (average n = 255) DZ data from the lower Nanaimo Group, Georgia Basin; by using MDAs to approximate TDAs, we assess the time-equivalency of lithostratigraphic units, and further resolve the depositional history of these strata. We also assess changes in sediment provenance within currently interpreted lithostratigraphic units by employing multi-dimensional scaling (MDS) of DZ populations^19^. From these data, we discuss the tectonic history of the Georgia Basin during deposition of the lower Nanaimo Group and demonstrate that DZ geochronology can be used to reliably resolve stratigraphic relations in structurally complex CMBs globally.

## Geological Setting and Study Area

**Figure 2:**
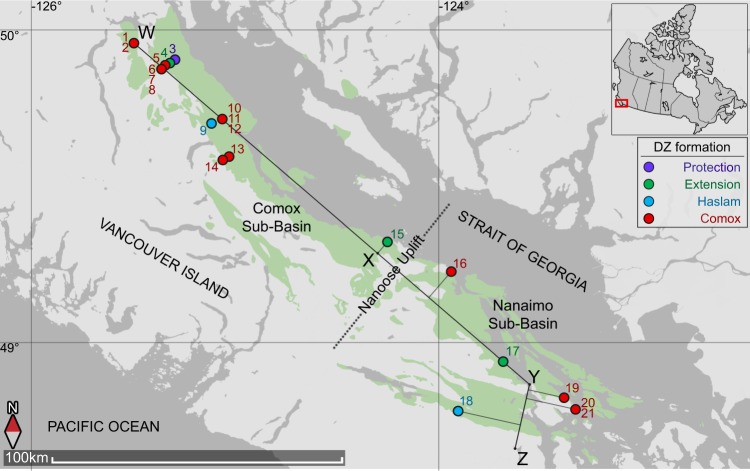
Distribution of the Nanaimo Group (green) on land. Strata mainly dip, and progressively young to the northeast. The circles numbered 1–21 refer to DZ samples, and the various colours indicate the lithostratigraphic unit from which samples were taken. The line of sections are a sequence stratigraphic cross-section of the Comox Sub-Basin (W to X; Fig. 6) and a chronostratigraphic reconstruction of the Georgia Basin (W to Z; Fig. 7). Inset figure shows the location of the study area in Canada.

The Nanaimo Group comprises a 4 km-thick sedimentary package of terrestrial to deep marine deposits which primarily outcrops across southeastern Vancouver Island, British Columbia, Canada. The main Nanaimo Group outcrops are separated into two sub-basins (Comox and Nanaimo) by a northeast trending topographic high known as the Nanoose Uplift (Fig. 2)^20^. Deposition of the Nanaimo Group and subsidence of the Georgia Basin was triggered by accelerated subduction of the Farallon and Kula Plates beneath North America^20^, mainly from Turonian to Maastrichtian time^20,21^.

Research on the Nanaimo Group has been motivated largely by economically-important coal deposits in the area, which were vital for shipping and railroads. Coal exploitation from the lower Nanaimo Group drove much of the colonial development on Vancouver Island. One-hundred and sixty years of study has led to the development and adoption of a lithostratigraphic framework for the group comprising 11 formations (Fig. 1)^1,22^. These formations are typically identified based on the position of fine-grained (mudstone-dominated) and coarse-grained (sandstone and conglomerate dominated) units relative to the basal nonconformity and to each other^1^. Formation ages are determined by molluscan and microfaunal biostratigraphy from outcrops that contain these fossils^21,23,24^. The 11 formations are grouped into the lower and upper Nanaimo Group. The lower Nanaimo Group comprises (in ascending order) the Comox, Haslam, Extension, Pender and Protection formations, and are predominantly terrestrial to shallow marine deposits. The upper Nanaimo Group comprises the Cedar District, De Courcy, Northumberland, Geoffrey, Spray and Gabriola formations, and are predominantly deep marine deposits (Fig. 1). The 11 formations that define the lithostratigraphy of the Nanaimo Group have been used to reconstruct the depositional history and internal architecture of the lower Nanaimo Group^22,25,26^ although this framework was not developed for such a purpose. While Nanaimo Group strata are generally homoclinal, strata in the Nanaimo sub-basin on Vancouver Island have undergone faulting and folding^1^ making local stratigraphic correlations difficult. Furthermore, many lower Nanaimo Group outcrops are unfossiliferous and their biostratigraphic ages are uncertain, particularly in the Comox Sub-Basin and in strata directly above the basal unconformity (Fig. 2)^23,24^. In consequence, highly diachronous strata are correlated together and time-equivalent strata are differentiated into separate formations^27^.

## Results

**Figure 3:**
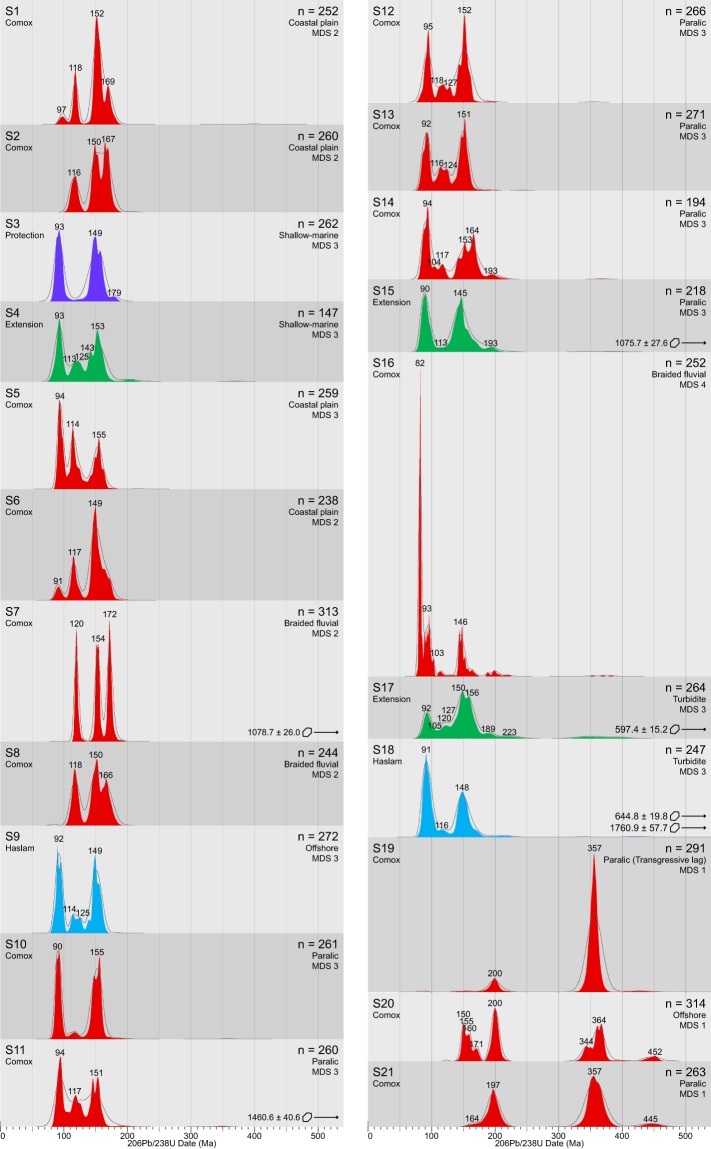
Normalized detrital zircon U-Pb spectra displayed as KDEs (filled) and PDPs (lines). Samples are stacked from northwest to southeast, and up section to down section. Significant age modes (grains in that mode represent >3% of the sample) are labelled. Below the sample name is the lithostratigraphic formation. The “n” values at the far right are the number of concordant grains measured in each sample; below the n values is the interpreted depositional environment (see Supplementary Data S1) and MDS group (Fig. 5). Y-axis is normalized probability. Plots are colour coded by formation using the same colour code as Fig. 2. Red = Comox Fm, Blue = Haslam Fm, Green = Extension Fm, and Purple = Protection Fm.

**Figure 4:**
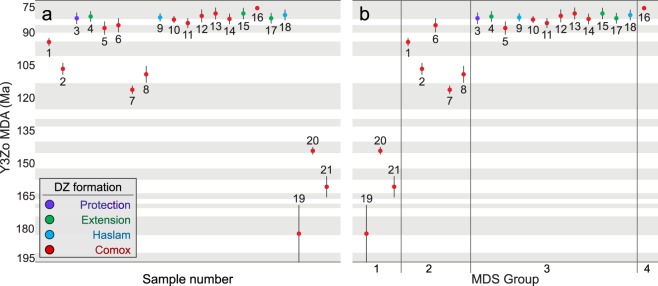
(**a**) Plot of MDAs derived from samples. The sample number is displayed below each datapoint, and MDA is on the y-axis. Coloured circles represent the MDA, and vertical lines show the 2σ uncertainty in ages. Maximum depositional ages are based on the youngest three grains overlapping at 2σ error (Y3Zo). The grey and white bands behind the samples shows the age range of the Geologic Age intervals (Aptian to Campanian). (**b**) The same data as (**a**), but organized by MDS Group (Fig. 5).

**Figure 5:**
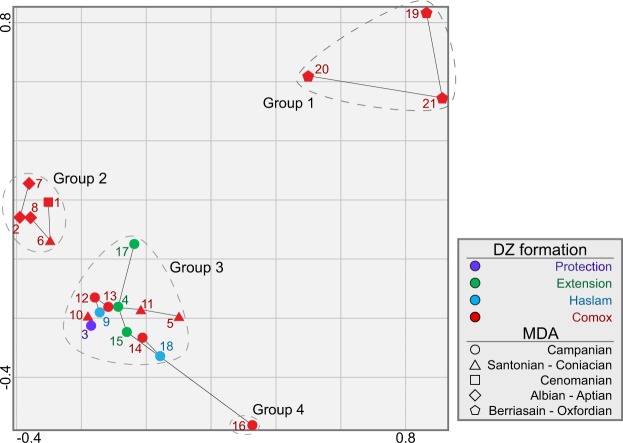
Non-metric Kuiper’s statistic MDS plot assessing similarities of DZ samples 1–21. Four MDS groups are defined based on nearest-neighbour analysis. Barring Group 4, all DZ samples in a group have nearest neighbours which map to another member of the group. Solid lines between samples indicate nearest neighbour relationships in n-dimensional space, not 2D space.

Detrital zircon grains from four of the five lower Nanaimo Group formations (Comox, Haslam, Extension and Protection) yielded 5,348 concordant dates from 21 samples (Fig. 3; see Supplementary Data S1). Maximum depositional ages derived from these samples (Fig. 4) range from the Toarcian (182.5 ± 13 Ma) to Campanian (78.6 ± 0.9 Ma). Multi-dimensional scaling of DZ age distributions (Fig. 5) yielded four groups (MDS Groups 1–4). Excluding MDS Group 1, all samples are dominated by <170 Ma grains (average = 93%) that originated from the associated arc^12,14^. Grains >170 Ma are attributed to the Sicker Group and Bonanza Arc, which form part of the underlying basement^28–32^ and the Descon Arc, which is another accreted terrane^33,34^. The main differences between MDS groups are summarized below (Table 1).

**Table 1:** Summary of salient differences between MDS Groups. Basement refers to the Bonanza Arc^28–30^ and the Sicker Group^31^, while “Other” refers to the Descon Arc^33,34^.

Source area	DZ mode (Ma)	MDS Group 1 ~160 to 145 Ma	MDS Group 2 ~110 to 85 Ma	MDS Group 3 ~85 to 80 Ma	MDS Group 4 79 Ma
Coast plutonic complex (associated arc)	82				O
	97-91		X	O	O
	120-113		O	X	
	156-145	X	O	O	O
	167-160	X	X	X	
Basement	171-169	X	X	X	
	200-189	O		X	
	364-344	O			
Other	452-445	O			
X – Some samples contain >3% mode			O – All samples contain >3% mode		

Multi-dimensional scaling Group 1 (Figs. 4, 5) comprises three samples (S19–21), all of which were collected from Comox Fm strata. Group 1 samples yield Jurassic (S19: 182 ± 13 Ma; S21: 160.5 ± 4.7 Ma; Fig. 4) and earliest Cretaceous (S20: 144.0 ± 1.6 Ma) MDAs. These samples are characterized by prominent modes at 364-344 Ma (average (avg) 49% contribution from this population; see Supplementary Data S1) and 200-197 Ma (avg 15%). Samples 20 and 21 contain an additional mode at 452-445 Ma (avg 4%). All 3 of these modes are rare in the other MDS groups.

Multi-dimensional scaling Group 2 comprises five samples (S1–2 and 6–8), all of which were collected from the Comox Fm. Group 2 samples yield Aptian (S7: 116.0 ± 1.8 Ma) to Coniacian (S6: 86.4 ± 3.1 Ma) MDAs. These samples are characterized by a prominent mode at 154-150 Ma (avg 40%), and variably sized modes at 172-166 Ma (avg 29%) and 120-116 Ma (avg 20%). Samples 1 and 6 also contain a small 97-91 Ma (avg 6%) mode.

Multi-dimensional scaling Group 3 comprises 12 samples, six of which were collected from the Comox Fm (S5 and 10–14), two from the Haslam Fm (S9 and 18), three from the Extension Fm (S4, 15 and 17) and one from the Protection Fm (S3). Group 3 samples from the Comox Fm yield MDAs from the Coniacian (S5: 87.7 ± 2.9 Ma) to the Campanian (S13: 81.0 ± 2.6 Ma), whereas samples from the Haslam, Extension and Protection formations only yield Campanian MDAs (S7: 83.2 ± 2.6 Ma to S15: 81.0 ± 2.5 Ma). All Group 3 samples are characterized by 158-145 Ma (avg 34%) and 94-91 Ma (avg 26%) modes. Group 3 samples also contain a 120-113 Ma (avg 8%) mode, and the proportion of this population decreases in samples with younger MDAs (S5: 87.7 ± 2.9 Ma and 22% to S3: 83.2 ± 2.5 Ma and 0%). As well, there are varying proportions of grains >170 Ma (S17: 19% to S9: 0%).

Multi-dimension scaling Group 4 comprises Sample 16, which was collected from the Comox Fm. Sample 16 yields a Campanian MDA (78.6 ± 0.9 Ma) and is characterized by a prominent 82 Ma mode (40%) that is absent from all other DZ samples. Smaller modes at 146 Ma (20%), 103 Ma (3%) and 93 Ma (25%) and are also present, as well as minor numbers of grains >170 Ma (6%).

## Discussion

### Considerations in the use of MDAs for stratigraphic analysis

Previous studies show that maximum depositional ages derived from convergent-margin basin DZ populations can approximate true depositional ages^5–8^ and our data support this observation. Our data also show that MDS groups generally correlate with MDA, and samples assigned to the same MDS group are broadly equivalent stratigraphically. However, both MDAs and MDS groups vary as a function of many factors, including depositional environment^35^, source area lithology^36^ and magmatic quiescence^6^. These physical aspects of the paleoenvironment impact DZ results and must be taken into consideration prior to their use in stratigraphic analysis.

**Figure 6:**
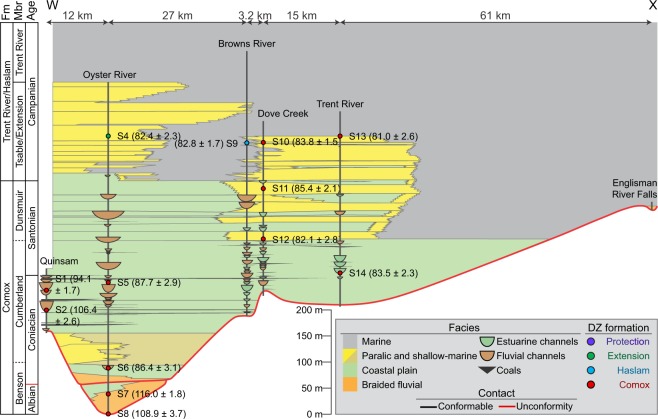
Interpreted sequence stratigraphy of the lower Nanaimo Group in the Comox Sub-Basin based on facies analysis, stratigraphic correlation and DZ MDAs (modified after Kent, et al.^27^). Strata are grouped into four depositional systems: braided-fluvial, coastal plain, paralic & shallow-marine, and marine. The position of DZ samples taken from the Comox Sub-Basin are shown on the vertical sections (vertical black lines). Acronyms: Formation = Fm; Member = Mbr.

Detrital zircon samples derived from marine environments (i.e., paralic, shallow marine, offshore and turbidite) are more likely to possess an MDA approximating TDA. Longshore drift in shoreface environments transports sediments along-strike significant distances^37^, incorporating DZ from multiple drainage basins^35^ and greatly increasing the chance of contribution from a broadly coeval source area. Conversely, MDAs derived from braided fluvial and coastal plain environments are less likely to approximate TDA than those from marine environments. For example, S2 and S1 have Albian (106.4 ± 2.6 Ma) and Cenomanian (94.1 ± 1.7 Ma) MDAs, respectively. However, sequence stratigraphic-based correlations of the host strata for the samples (Fig. 6) indicate that both S2 and S1 are probably time-equivalent with Coniacian strata rather than with Albian and Cenomanian strata as their MDAs suggest^27^. An absence of broadly-coeval DZ (i.e., Coniacian-aged DZ) in both samples is attributed to the depositional environment of the host strata, which is interpreted as terrestrial to coastal plain^27^. The DZ age spectra generated for S2 and S1 are interpreted to reflect the ages of rocks in the limited catchment areas of their associated rivers, which must have lacked DZ broadly coeval with their TDA. Alternatively, the strata from which S2 and S1 are derived can be as old as their MDAs suggest, and the sequence stratigraphic correlations presented in Kent, *et al*.^27^ may require revision to reflect this. Resolving this uncertainty will require higher-resolution sampling of strata in the NW extent of the Georgia Basin.

Strata comprising transgressive lags (S19) are formed through the cannibalization of older strata, and hence contain DZ age spectra which are reflective of older sedimentary deposits rather than broadly-contemporaneous non-lag deposits. Although S19 yields a Toarcian MDA (182 ± 13 Ma; Fig. 4), it also includes three significantly younger grains (81.7 ± 3.7 Ma, 91.5 ± 3.3 Ma, 138.8 ± 5.0 Ma; Supplementary Data S1). Consequently, S19 is considered to have an MDA that does not approximate TDA, and the transgressive lag from which S19 was derived is interpreted to be Campanian based on previous stratigraphic correlations^38^, which is also in agreement with the age of the youngest DZ grain analyzed. In addition to the paucity of Cretaceous-aged DZ, S19 has sizable contributions from populations with 357 Ma (74%) and 200 Ma (7%) modes, characteristic of MDS Group 1 samples (49% and 15%, respectively). This is interpreted to reflect the transgressive reworking of a sandstone with similar age modes to S21, and results in nearest-neighbour analysis assigning S19 to MDS Group 1 rather than 3.

In many deep-marine environments, such as turbidite channels and levees (S17 and 18), sediments from multiple source areas are captured, mixed and sequestered. This can result in samples from turbidite strata containing DZ populations that are absent from temporally-equivalent non-turbidite samples^35^. For example, amongst samples possessing Santonian to Campanian MDAs (S3, 4 and 9–18), there is significantly higher proportions of basement DZ (age >170 Ma) from turbidite strata (avg 10%) than other environments (avg 4%). These differences in DZ age populations can cause turbidite samples to become spatially separated on an MDS plot (more dissimilar) from temporally equivalent non-turbidite samples. For example, S17 possesses 155–145 Ma (64%) and 94–91 Ma (13%) modes which deviate greatly from the averages of other Group 3 samples (30% and 27%, respectively), and more closely resemble proportions found in Group 2 samples (40% and 6%, respectively; Fig. 5). In fact, other methods of calculating dissimilarity, such as the Kolmogorov-Smirnov test, causes nearest-neighbour analysis to assign S17 to MDS Group 2 rather than 3.

Although using MDAs as TDAs to determine the stratigraphic position of samples separated by 10s of Myr is generally justifiable, this technique is not appropriate for distinguishing depositional architectures at the bedset-scale. In Mesozoic and older CMBs, modern DZ analytical techniques generate datasets with MDAs yielding uncertainties in the millions of years^39^. This is exemplified in this study, in that the average 2σ uncertainty of MDAs is 2.4 Myr, which is well below the resolution necessary to resolve depositional architectures of Campanian-aged samples (Fig. 4). Despite the average 2σ uncertainty of MDAs being lower in younger CMBs, there is a lag time associated with zircon crystallization, arc batholith exhumation, erosion and deposition which will dictate how closely MDA approximates TDA. While Bayesian statistics can provide some inferences on TDA^40^, the precision of such calculations depend on *a priori* information such as sediment accumulation rates and known stratigraphic relationships.

### Diachroneity of lower nanaimo group lithostratigraphic formations

After accounting for limitations in the application of DZ analysis in stratigraphic studies, the DZ data presented herein still reveal a significantly more complicated depositional history for the lower Nanaimo Group, Georgia Basin, than has previously been recognized. Viable MDAs (i.e. all MDAs barring S1, 2 and 19) demonstrate that individual formations presently assigned to lower Nanaimo Group strata are not always time-correlative, and strata assigned to the same formation do not always share source regions. Specifically, MDS analyses demonstrate that multiple sediment sources of varying ages existed for the Georgia Basin and that they evolved throughout time (Table 1). The distribution of sediments from these sources do not correlate by formation and is better predicted by MDA and geographic position.

The Comox Fm is the most problematic formation in terms of age and lithostratigraphic assignment. The Comox Fm is defined lithostratigraphically as the first coarse clastic unit directly overlying the basal nonconformity^1^ and is assigned a Turonian to Santonian depositional age based on molluscan biostratigraphy^1,21^. In this study, viable Comox Fm MDAs (Fig. 4) range from the Jurassic (Oxfordian (S21)) to the Upper Cretaceous (Campanian (S12, 13 and 16)), and Comox Fm DZ samples share affinities with all 4 MDS groups (Fig. 5); this reflects variable sediment source areas throughout time and space. These results indicate that the Comox Fm is a highly diachronous depositional unit, and contains previously unrecognized disconformities^27^. Comox Fm coarse clastics span deposition of the entire lower Nanaimo Group, and reflect a continued and slow drowning of the basal nonconformity as the Georgia Basin underwent multiple phases of subsidence, deposition and termination throughout the Cretaceous.

Overlying the Comox Fm are the Haslam, Extension and Protection formations, all of which yield similar MDAs and group into MDS Group 3 (Figs. 4 & 5). Some Comox Fm samples also show similar MDAs and age distributions (MDS Group 3) to the three overlying formations. The similar MDAs and MDS groups of some Comox and all Haslam, Extension and Protection formation samples is attributed to near-contemporaneous deposition of these stratal units due to very high sedimentation rates. Presently, deposition is interpreted to have extended from the Santonian to the end of the lower Campanian (a period of >10 Myr)^21,24^. However, CMBs commonly experience very rapid sediment accumulation rates (e.g., >1,650 m Myr^−1^ in the Kumano Forearc Basin, Japan^41^). As the host strata for MDS Group 3 samples are up to 1,200 m thick^1,27^, this raises the possibility that these strata represent sediments that largely accumulated in a much short period of time. For example, using accumulation rates from the Kumano Forearc, 1,200 m of lower Nanaimo Group strata represents 727 Kyr. The result of this would be MDAs that overlap within uncertainty, and age distributions that reflect similar sediment sources contributing sediment to the Georgia Basin during deposition of these strata; i.e., the same sediment sources and associated catchments existed for a ~1 Myr period during deposition of the Haslam, Extension and Protection formations. This view is supported through recent sequence stratigraphic reconstruction of the lower Nanaimo Group in the Comox Sub-Basin, wherein lithoformations that were previously interpreted as representing sequential deposition^25^ are instead shown to reflect contemporaneous deposition and lateral facies variability (S4, 9, 10 and 13; Fig. 6)^27^.

### A revised early evolution for the Georgia Basin

**Figure 7:**
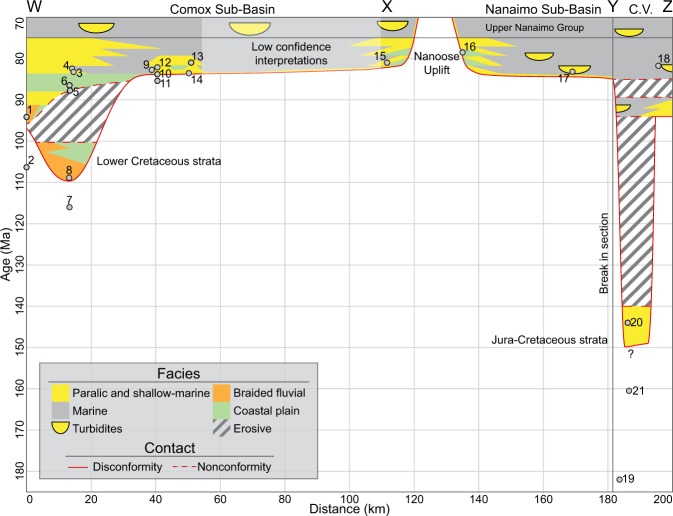
Interpreted chronostratigraphy of the lower Nanaimo Group shown as a Wheeler Diagram for the Georgia Basin. Orange units are conglomerate dominated, green units are mudstone dominated, yellow units are sandstone dominated, and gray units are shale and mudstone dominated. The white area below the red line at the base of the Nanaimo Group represents both non-deposition and pre-Nanaimo Group strata (e.g., Jurassic volcanics) while the gray-hatched areas represent non-deposition / erosion within the Nanaimo Group. Circles with numbers indicate MDAs derived from DZ samples and sample numbers are given – note that some MDAs are interpreted to pre-date the basal unconformity. The boundary between the lower and upper Nanaimo Groups is placed in the Campanian to reflect results from biostratigraphy^21,24^. Acronyms: C.V. = Cowichan Valley.

The early evolution of the Georgia Basin has been interpreted based on lithostratigraphy and biostratigraphy of the lower Nanaimo Group^22,25,26^, which suggested that basin subsidence began during the late Santonian/early Campanian^20^. An alternate early evolution history derived from sequence stratigraphic correlations^27^ and DZ geochronology is that the Georgia Basin underwent multiple phases of subsidence, deposition and non-deposition/erosion, and that allogenic influences controlling sedimentation and sediment accumulation varied through time and space. Variable MDAs and age distributions in the Comox Fm suggest the subsidence and uplift history of the Georgia Basin was complex, and multiple potential hiatuses exist in the Comox Sub-Basin (Turonian-Santonian hiatus) and the Nanaimo Sub-Basin (Jurassic-Turonian hiatus; Fig. 7). The Haslam, Extension and Protection formations show greater similarity in terms of depositional age, DZ age distributions and source areas which persisted throughout deposition of these strata. These similarities are interpreted to record rapid subsidence of the Georgia Basin during the Campanian. Together the DZ results suggest a more complicated initiation and subsidence history to the Georgia Basin than has previously been described and potentially the preservation of both Jurassic (S21) and Lower Cretaceous (S7, 8 and 20) strata in a basin previously described as forming exclusively in the Upper Cretaceous (Fig. 7). Confirmation of the existence of older strata can further resolve the Baja BC hypothesis, as focused studies of potential Jurassic and Lower Cretaceous strata can provide constraints on the location of the Baja BC block relative to North America prior to the Upper Cretaceous.

## Conclusions

Twenty-one DZ samples from the lower Nanaimo Group, Georgia Basin, Canada were analyzed to test and refine the established depositional history and stratigraphy for the basin, and to consider the utility of DZ MDA analysis in CMBs. Our results indicate that the depositional architectures and evolution of the Georgia Basin cannot be accurately constrained using the existing lithostratigraphic framework. Viable Comox Fm MDAs range over 80 Myr (Fig. 4), and age distributions of Comox Fm samples vary significantly across the Georgia Basin (Fig. 5) despite being correlated together lithostratigraphically. As well, samples collected from the three formations overlying the Comox Fm reveal MDAs that reflect rapid sediment accumulation and coeval deposition rather than sequential deposition, and together these results reveal a more complicated initiation to the Georgia Basin than has previously been described. We hypothesize that during its early stages of formation, the Georgia Basin experienced multiple phases of subsidence and uplift, leading to the development of a diachronous basal unconformity with significant topography, and internal disconformities spanning millions of years; this is consistent with observations made in other forearc basins, and suggests a common initiation mechanism for forearc basins.

This study also highlights how MDAs from DZ geochronology can be used to establish and refine basin-scale stratigraphic frameworks, particularly in CMBs. This technique is useful for resolving long-distance stratigraphic correlations and identifying potential disconformities, particularly in localities where strata is unfossiliferous and/or structurally complex. However, considerations should be given to the paleo-depositional environment to evaluate the reliability of calculated MDAs in approximating TDA. Specifically, DZ samples taken from strata deposited in braided fluvial and coastal plain environments yield less reliable MDAs than those collected from strata deposited in paralic, shallow marine, offshore and turbidite environments. Furthermore, DZ MDAs are too imprecise to resolve depositional architectures at the bedset-scale, due to both analytical error and uncertainties with depositional lag time. Nevertheless, DZ MDAs represent a powerful technique for resolving stratigraphy in CMBs, especially when ground-truthed through facies analysis and biostratigraphy.

## Methods

Twenty-one DZ samples were collected and analysed at high-n (n = 147 to 314) from lower Nanaimo Group strata in outcrops and core along a 200 km transect through the Nanaimo and Comox sub-basins (Fig. 2; see Supplementary Data S1). Samples represent four of the five previously established lower Nanaimo Group lithostratigraphic formations. Each DZ sample was assigned a lithostratigraphic formation by comparison to published lithostratigraphic schemes and geological maps, and by comparing sedimentological and ichnological characteristics to existing formation descriptions. Depositional environments for sampled strata are derived from Jones^42^, Jones, *et al*.^43^ and Kent, *et al*.^27^, and these are used to evaluate the impact of sediment transport processes in each environment on DZ age distributions. Strip logs displaying the location of DZ samples in core or outcrop are presented in Supplementary Figure S2.

### Sample processing

Three to five kg of sandstone were collected for each sample. Samples were broken up, cleaned with a scrub brush and then treated with 30–50% hydrogen peroxide to remove moss, algae and other organic material. Detrital zircon were isolated using standard gravimetric and magnetic mineral separation techniques at Simon Fraser University that included: 1) mechanically crushing and disk milling the samples, 2) gravitational separation using a Wilfley table and heavy liquids (methylene iodide), 3) using a rare earth magnet and LB-1 Frantz® Magnetic Barrier Laboratory Separatory to remove the most magnetic grains from the heavy mineral concentrate. Samples rich in pyrite were also immersed in nitric acid .

Epoxy mounting and LA-ICP-MS U-Th-Pb dating was conducted at the Calgary Geo- and Thermochronology Lab, University of Calgary, Calgary, Alberta, Canada and the Arizona LaserChron Center, University of Arizona, Tucson, Arizona, USA. Processed LA-ICP-MS data are presented in Supplementary Data S1. Samples sent to the Geo- and Thermochronology lab at the University of Calgary were further refined using the Franz magnetic separator to isolate DZ. Detrital zircon in samples processed at the University of Arizona were hand-picked from the concentrate.

Samples processed at the Calgary Geo- and Thermochronology Lab were ablated using an ASI Resochron™ 193 nm excimer laser ablation system which incorporated a Laurin Technic M-50™ dual volume chamber. Isotopic signal intensities were measured using an Agilent 7700 quadrupole-ICP-MS. The ablation sequence employed a reference material-unknown bracketing procedure, with a measure of a FC1 zircon^44^ every 20 unknowns. Zircon reference materials FCT^45^, Temora 2^46^, 91500^47^ and internal GSC reference 1242 from the Lac Flechette syenite were employed to validate the results and to assess uncertainties. Data reduction was facilitated by the lolite™ (V2.5) software package^48^ using the VizualAge data reduction scheme^49^. Finally, analyses with a probability of <1% concordance were eliminated from DZ datasets. Detailed analytical procedures for the Calgary Geo- and Thermochronology Lab are available in Matthews and Guest^50^.

Samples processed at Arizona LaserChron Center were ablated using a 193 nm Teledyne Analyte G2™ ArF laser ablation system, which was equipped with a HelEx-2™ dual-volume chamber. Isotopic signal intensities were measured using a Element 2 Single Collector-ICP-MS. Sri Lanka F^51^, FC1^44^ and R33^46^ zircon were used as the calibration reference materials to correct for elemental and isotopic fractionation. FCT^45^, 94-35^52^, Plešovice^53^, Temora 2^46^, Peixe, 91500^47^, Oracle, QGNG^54^, Tan-BrA and OG-1^55^ zircon were used as validation reference materials. Data reduction was conducted using AgeCalc, an Excel-based data processing routine^56^. Detailed Arizona LaserChron Center procedures are available in Gehrels, *et al*.^57^.

### Data processing

Data from LA-ICP-MS were imported into Density Plotter^58^ and used to generate age distributions which are presented as kernel-density estimations (KDEs). Kernel-density estimations are a statistical visualization technique that assumes a Gaussian distribution around each measured age but does not account for analytical uncertainties^58^. Probability density plots (PDPs) were also generated and PDPs account for analytical uncertainties around each measured age^58^.

Age modes were selected using Age Pick 2010 and modes are displayed on the age distribution diagram (Fig. 3) if the associated population represents more than 3% of the sample. Modes selected by Age Pick 2010 that are less than 5 Myr apart were merged, and a new mode was calculated by taking the weighted-average between the original modes. Contributions of populations from each mode to each DZ sample are presented in Supplementary Data S1.

Maximum depositional ages were taken to approximate TDAs in this study. Strata with similar MDAs were inferred to be broadly temporally equivalent^5–8^ and strata with different MDAs were inferred to be temporally inequivalent. Maximum depositional ages were calculated by taking the weighed-average of the three youngest grains overlapping at 2σ uncertainty (Y3Zo) using the “Weighted Average” function of Isoplot 4.0^59^. The use of Y3Zo is considered an acceptable compromise between using the youngest single grain (YSG), which yields an MDA closest to true depositional age but is subject to Pb-loss effects, and using the youngest three or more grain cluster overlapping at 2σ uncertainty (Y3G + @ 2σ), which is statistically reliable but generates overly-conservative MDAs^6,39^. Alternative MDA calculations using YSG and Y3G + @ 2σ are presented in Supplementary Data S1. All dates used in MDA calculations were obtained from ^206^Pb/^238^U ratios.

A multi-dimensional scaling (MDS) plot was generated, which sorted DZ samples into groups (MDS Groups) based on nearest neighbour analysis. Samples assigned to the same MDS group (excluding S19) were interpreted to be broadly stratigraphically equivalent (i.e., bounded by the same major disconformities), while samples that were grouped apart were interpreted to be stratigraphically non-equivalent (i.e., separated by one or more major disconformities). With the exception of MDS Group 4, DZ samples sharing a group have nearest neighbours that map to another member of the group. The MDS plot was generated with detritalPy^60^, a program which uses the Kuiper statistic (V_max_) as a dissimilarity metric for MDS. On the resulting plot, samples with similar age populations were grouped together, and samples with dissimilar age populations were grouped apart. Dissimilarity measures generated using Kuiper’s test and the Kolmogorov-Smirnov test, along with nearest neighbour and second nearest neighbour analyses, are presented in Supplementary Data S1.

## Supplementary information

Supplementary Dataset 1

Supplementary Dataset 2

## Data Availability

The datasets generated and analyzed during this study are included in this published article (and its Supplementary Information files).
