# Supplementary material for: Resolving the Architecture and Early Evolution of a Forearc Basin (Georgia Basin, Canada) Using Detrital Zircon
Source: Sci Rep. 2019 Oct 25;9:15360. doi: 10.1038/s41598-019-51795-5 (PMC6814742; doi:10.1038/s41598-019-51795-5)

**RESOLVING THE ARCHITECTURE AND EARLY EVOLUTION OF A FOREARC BASIN (GEORGIA BASIN, CANADA) USING DETRITAL ZIRCON**

Chuqiao Huang^*1^, Shahin E. Dashtgard^1^, Bryan A.P. Kent^1^, H. Daniel Gibson^1^, William A. Matthews^2^

^1^Applied Research in Ichnology and Sedimentology (ARISE) Group, Department of Earth Sciences, Simon Fraser University, Burnaby, B.C., Canada V5A 1S6

^2^Department of Geoscience, University of Calgary, Calgary, AB, Canada T2N 1N4

*email: chuqiaoh@sfu.ca

**Supplementary Figures S2 – Locations of DZ samples in vertical section**

**Quinsam Coal Mine Core**

The Quinsam Coal mine section is based on QU-11-13, a core drilled near Campbell River, Vancouver Island. The surface coordinates are 49.948703° N, 125.448569° W. Detrital zircon samples 1 and 2 were collected from it. The Y-axis indicates increasing depth below surface (0 m; top right of well log), where surface represents the youngest strata in the cored interval. Paleoenvironment interpretations and additional information can be found in Kent, et al. ^27^.


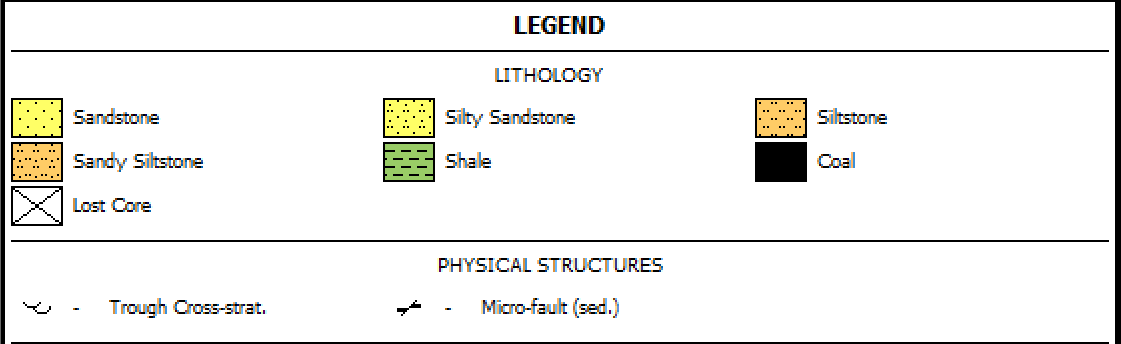


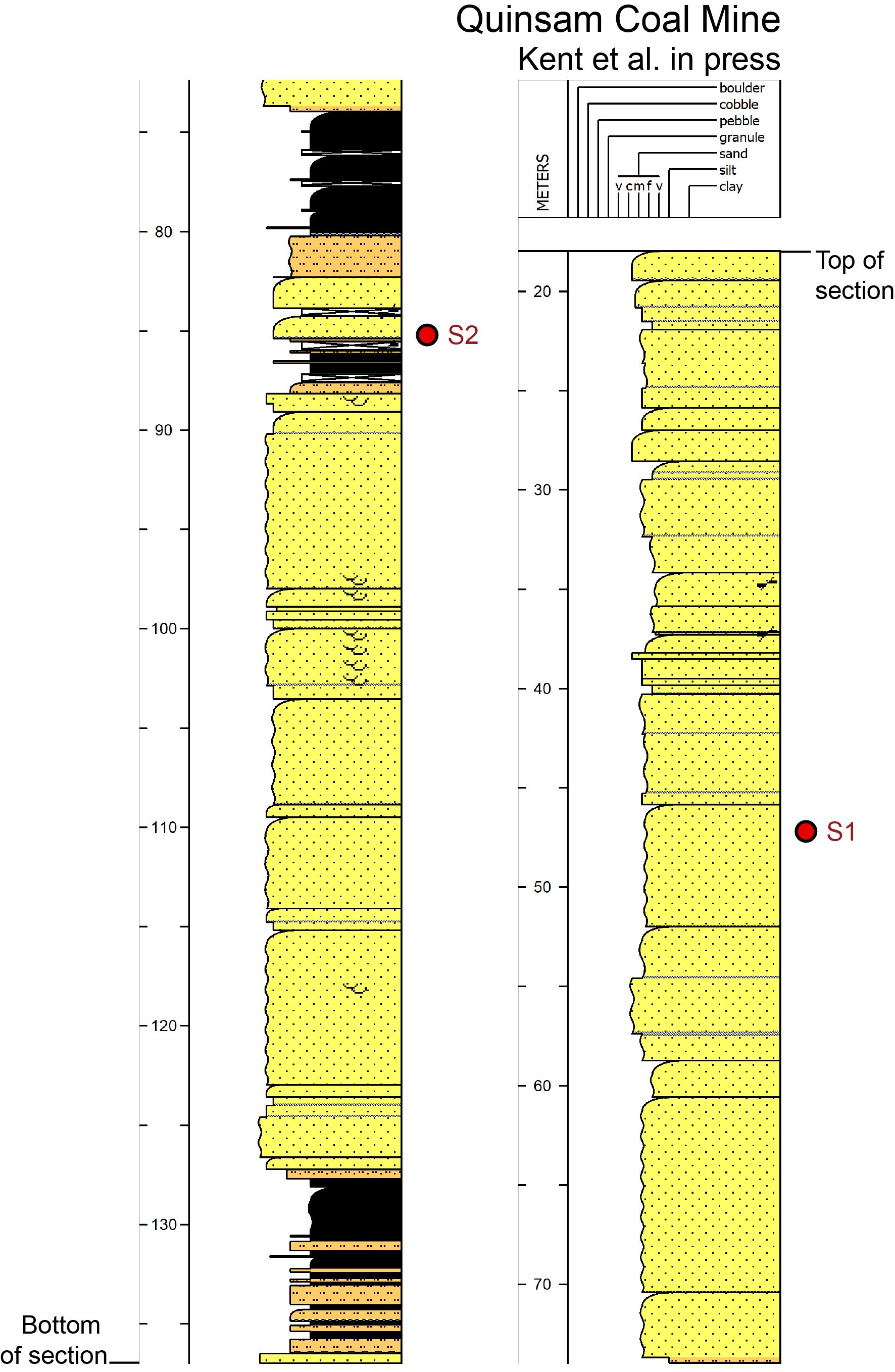


**Oyster River Outcrop**

The Oyster River outcrop runs along the Oyster River on Vancouver Island, B.C., Canada. The base of section is at 49.86496° N, 125.31871° W and the top of section is at 49.89521° N, 125.22430° W. Detrital zircon samples 3, 4, 5, 6, 7 and 8 were collected from along the Oyster River, although sample 3 was collected beyond the extent of the section. The Y-axis indicates stratigraphic height above the base of section (approximately 1 m; bottom left) where the base of section represents the oldest strata of the Nanaimo Group along the Oyster River. Paleoenvironment interpretations and additional information can be found in Kent, et al. ^27^.


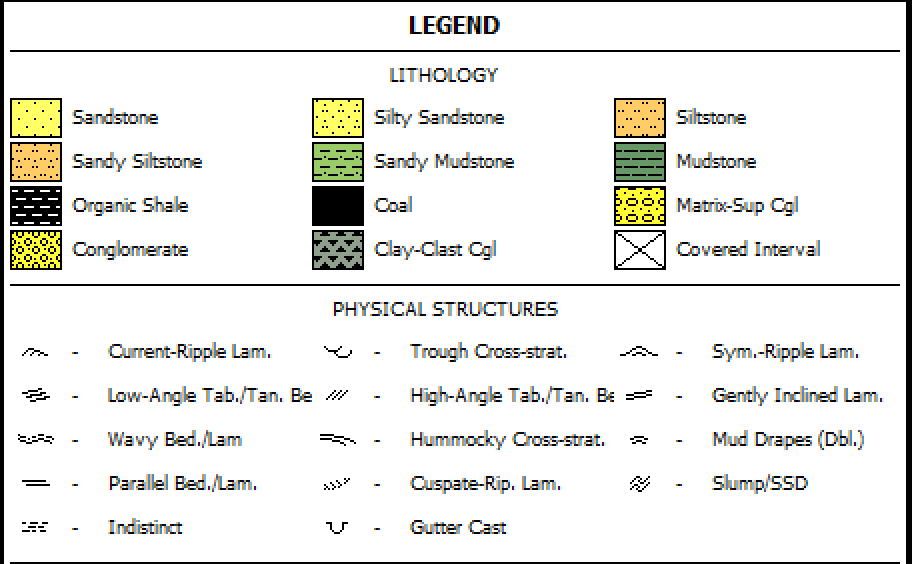


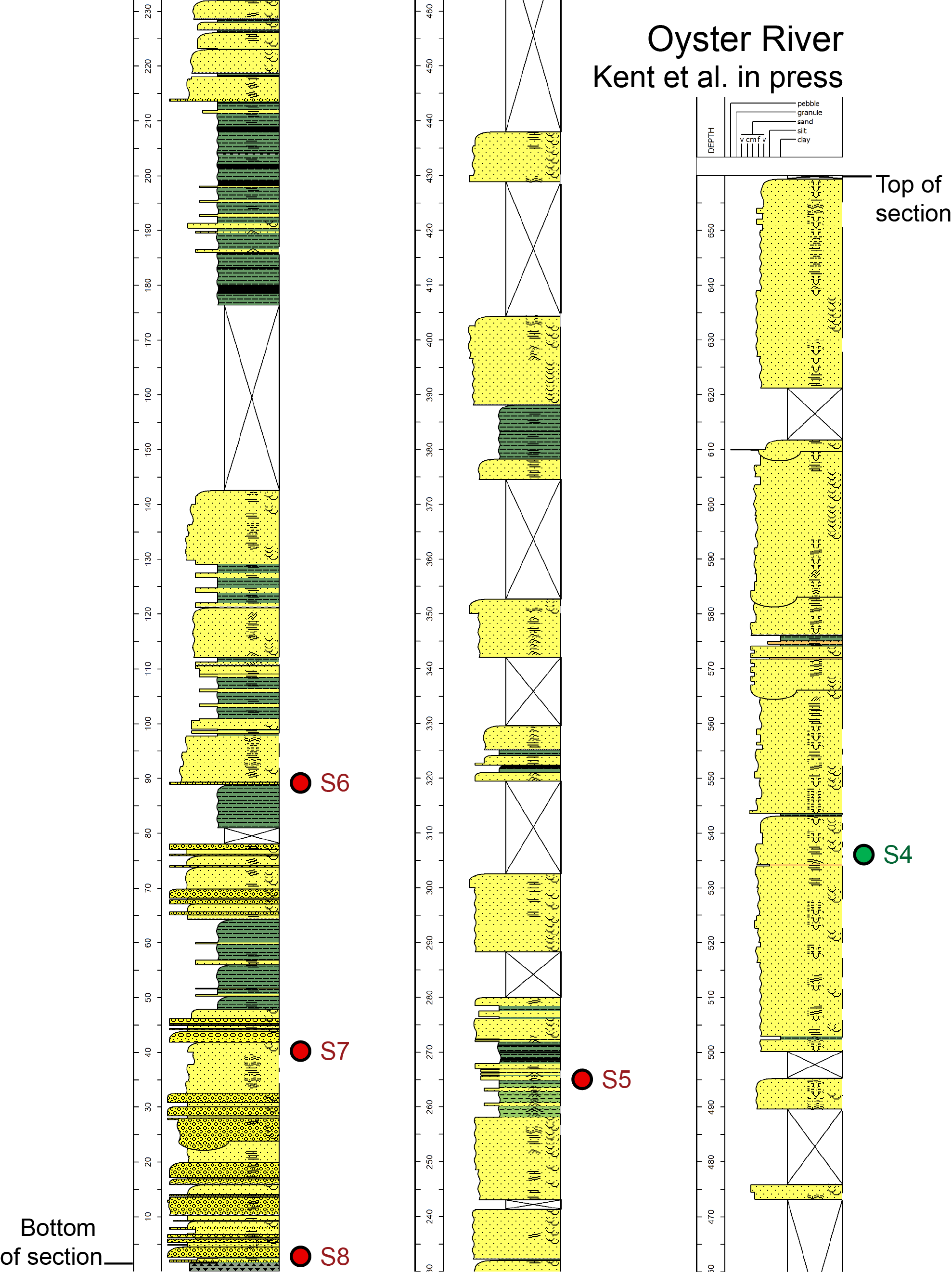


**Browns River Outcrop**

The Browns River outcrop runs along the Browns River on Vancouver Island. The base of section is at 49.68916° N, 125.05077° W and the top of section is at 49.69035° N, 125.12851° W. Detrital zircon sample 9 was collected from it. The Y-axis indicates stratigraphic height above the base of section (approximately 3 m; bottom left) where the base of section represents the oldest strata of the Nanaimo Group along the Browns River. Paleoenvironment interpretations and additional information can be found in Kent, et al. ^27^.


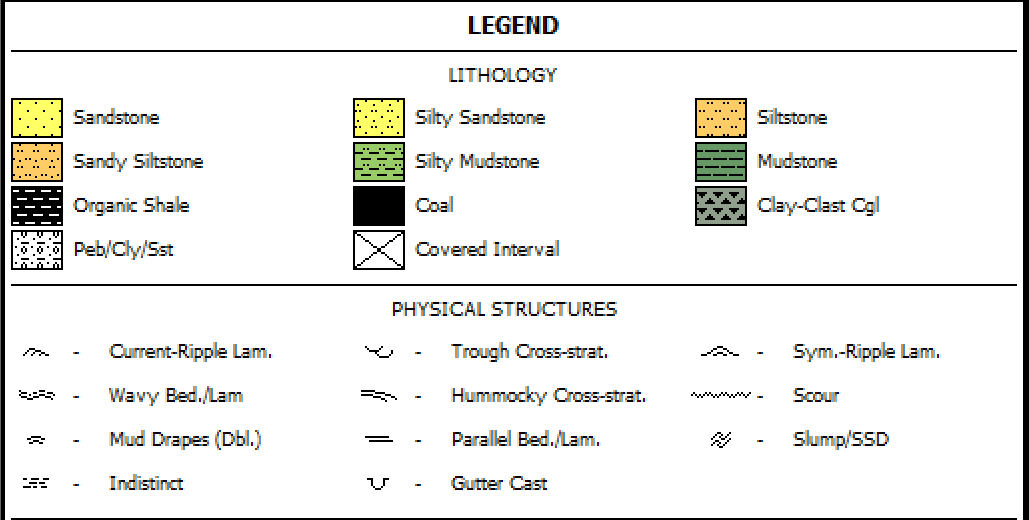


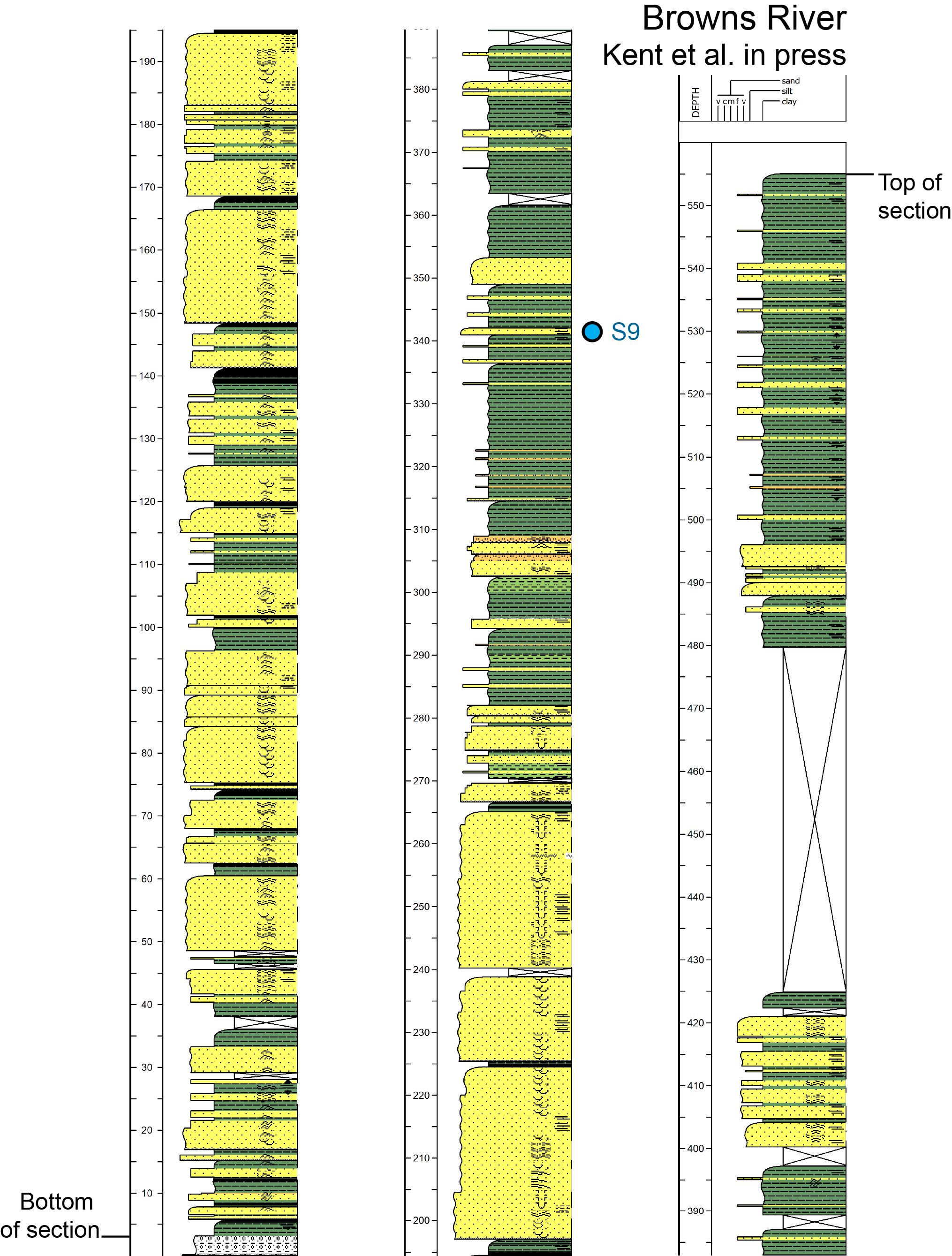


**Dove Creek Core**

The Dove Creek section is based on D2-A, a core drilled near Courtenay, Vancouver Island. The surface coordinates are 49.70579° N, 125.02383° W. Detrital zircon samples 10, 11 and 12 were collected from it. The Y-axis indicates increasing depth relative to the land surface (0 m). The start of the cored interval is ~150 m and the youngest strata of the Nanaimo Group in the cored interval occur at approximately 175 m. Paleoenvironment interpretations and additional information can be found in Kent, et al. ^27^.


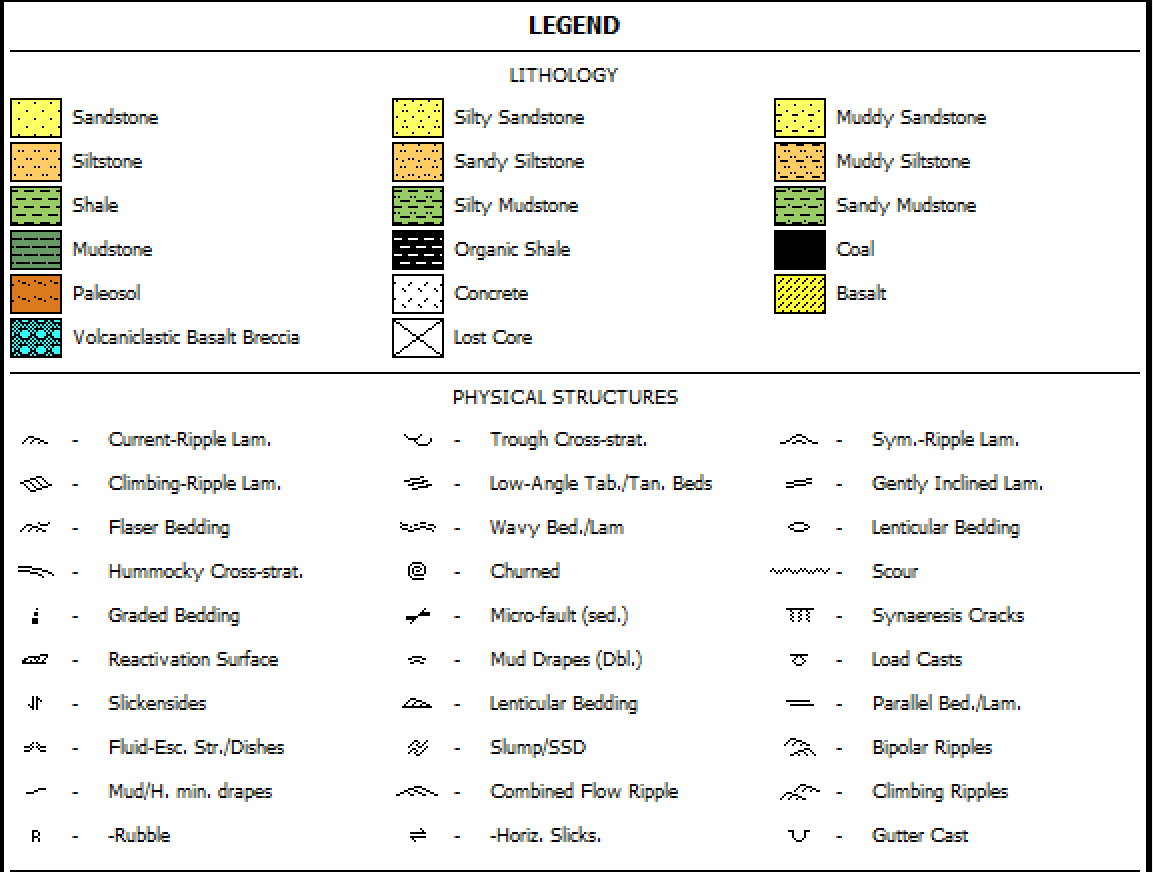


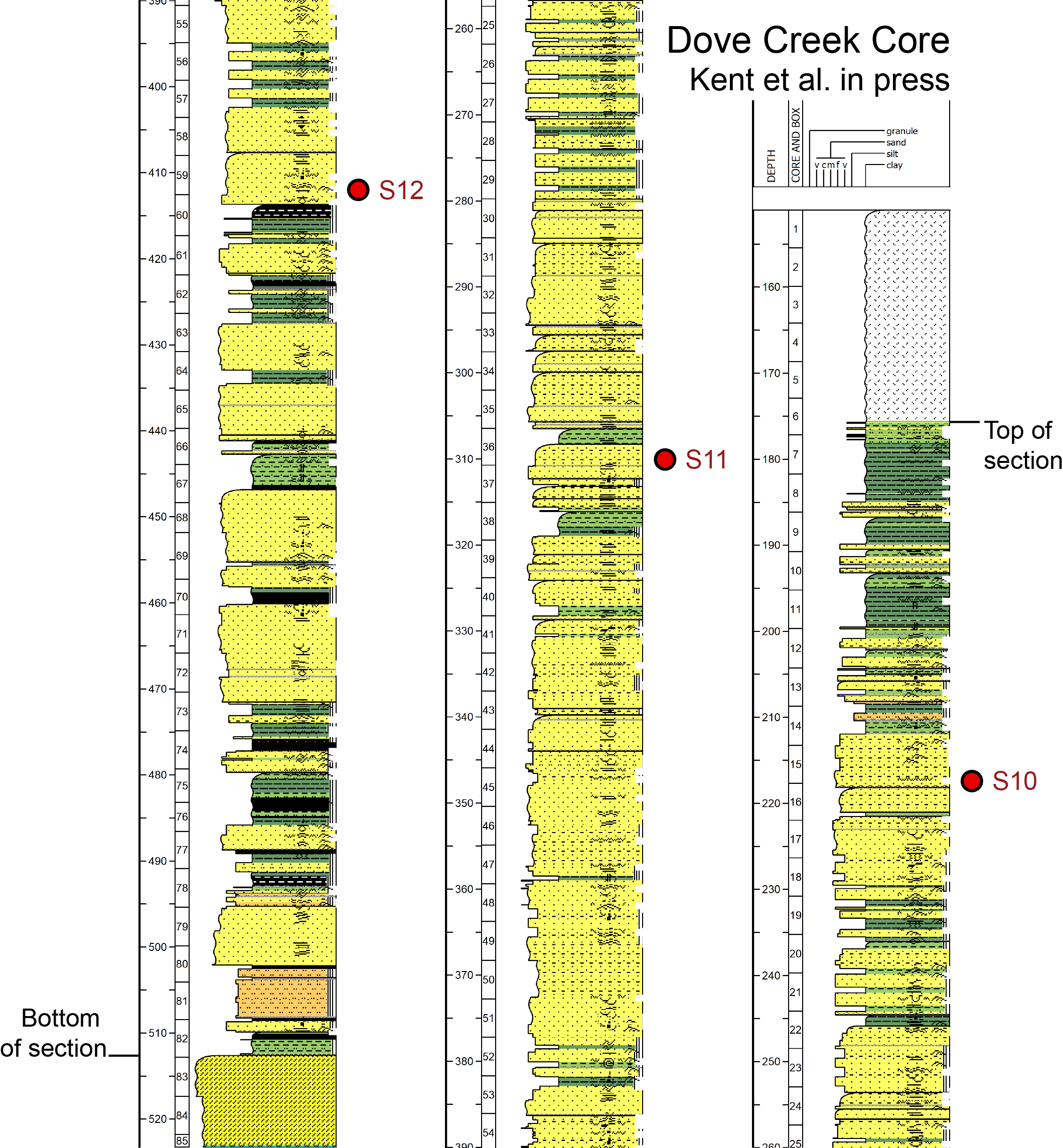


**Trent River Outcrop**

The Trent River outcrop runs along the Trent River on Vancouver Island. The base of section is at 49.57817° N, 125.02532° W and the top of section is at 49.59377° N, 124.98992° W. Detrital zircon samples 13 and 14 were collected from it. The Y-axis indicates stratigraphic height above the base of section (approximately 5 m; bottom left) where the base of section represents the oldest strata of the Nanaimo Group along the Trent River. Paleoenvironment interpretations and additional information can be found in Kent, et al. ^27^.


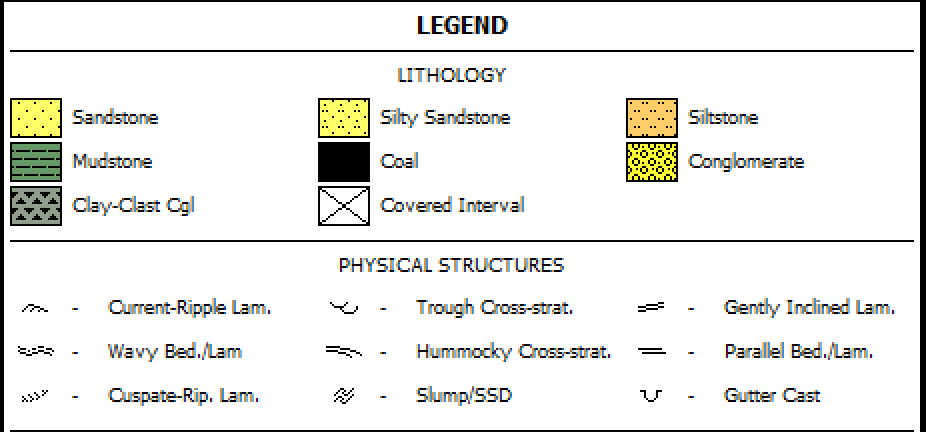


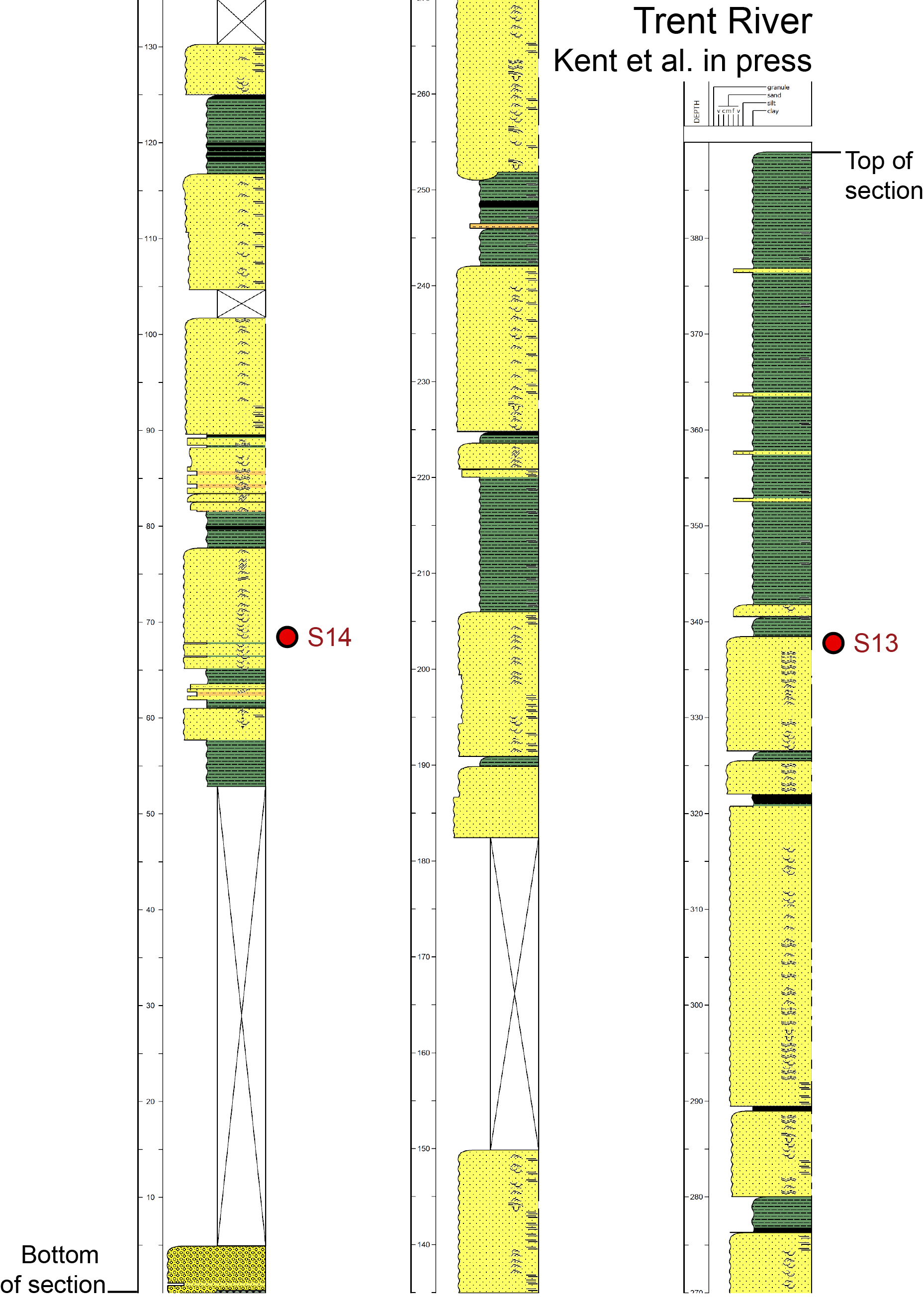


**Wall Beach Outcrop**

The Wall Beach outcrop is located in Parksville, Vancouver Island. The base of section is at 49.3127° N, 124.24262° W. Detrital zircon sample 15 was collected from it. The Y-axis indicates stratigraphic height above the base of section (0 m; bottom left) where the base of section represents the oldest strata of the Nanaimo Group exposed at the Wall Beach outcrop location. Paleoenvironment interpretations and additional information can be found in Jones, et al. ^38^.


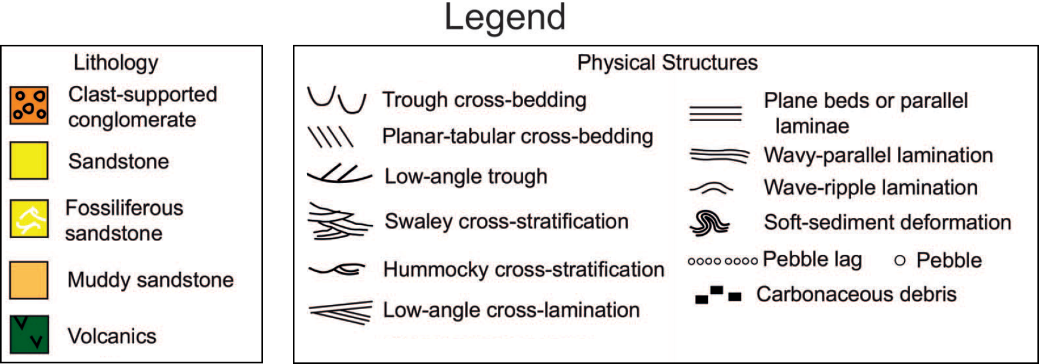


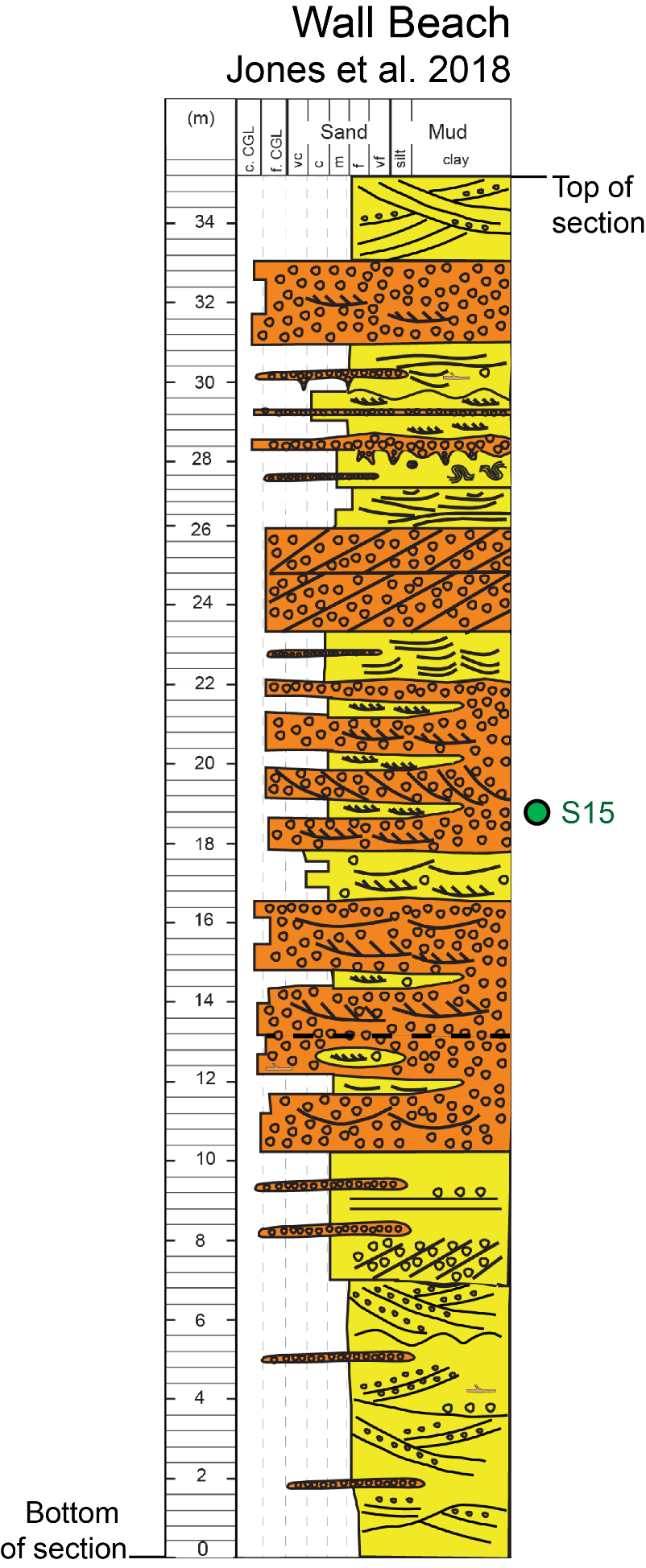


**Departure Bay Outcrop**

The Departure Bay outcrop is located in Nanaimo, Vancouver Island. The base of section is at 49.21439° N, 123.94049° W. Detrital zircon sample 16 was collected from it. The Y-axis indicates stratigraphic height above the base of section (approximately 1 m; bottom left) where the base of section represents the oldest strata of the Nanaimo Group. Paleoenvironment interpretations and additional information can be found in Jones, et al. ^38^.


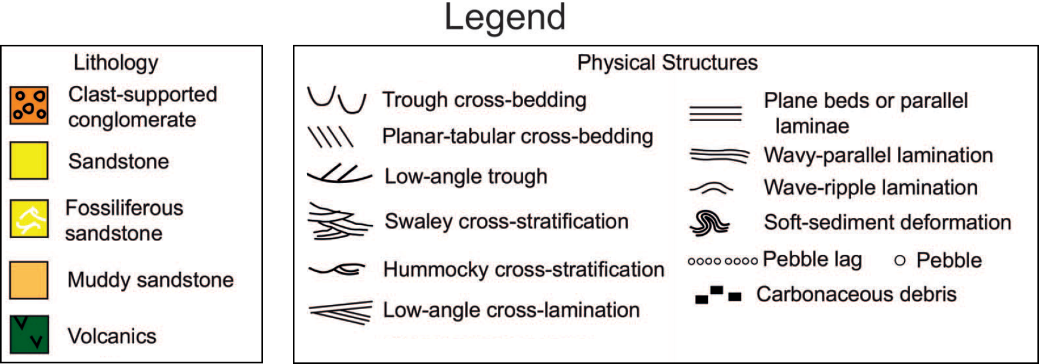


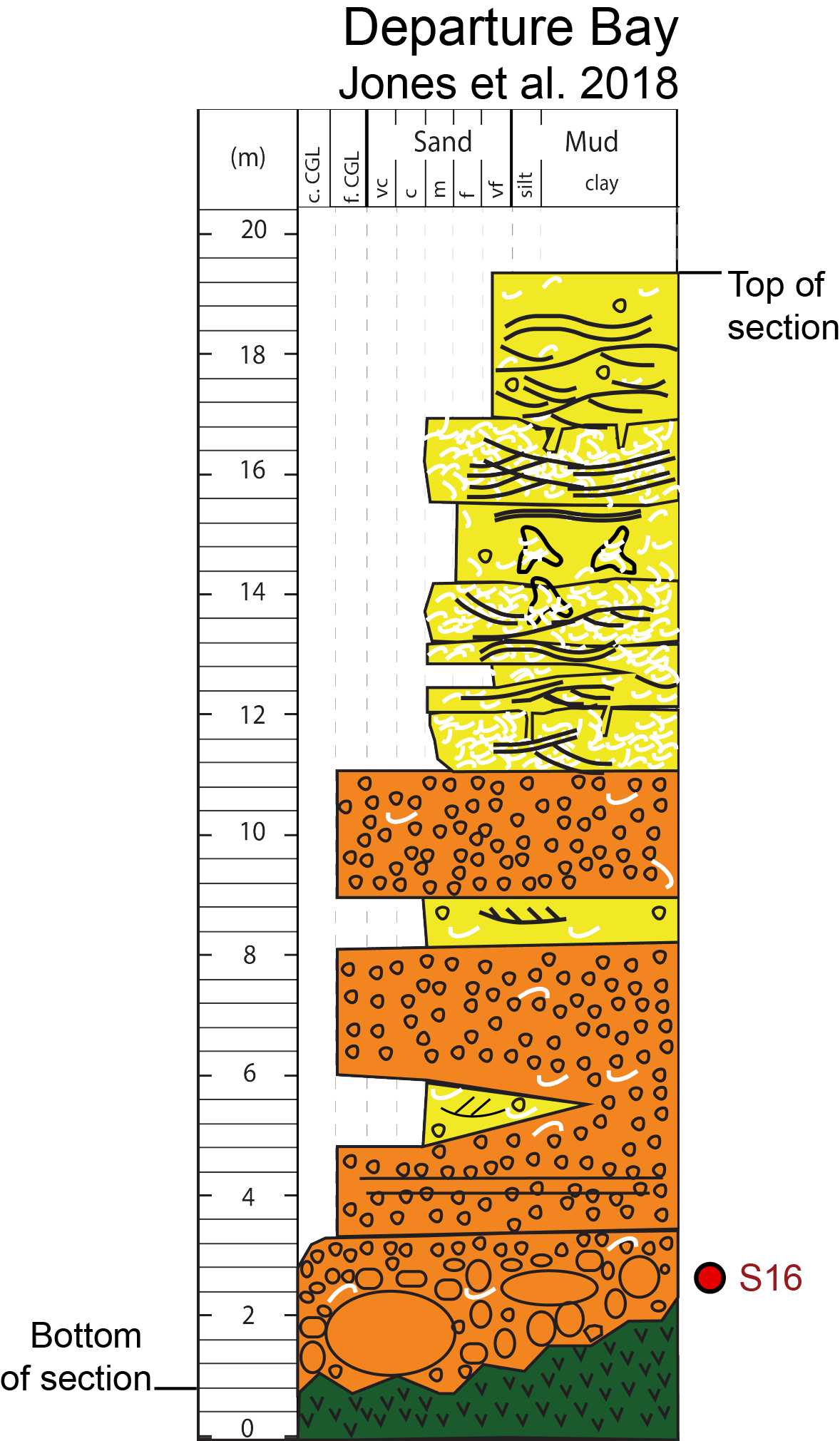


**Bare Point Outcrop**

The Bare Point outcrop is located near Chemainus, Vancouver Island. The base of section is at 48.92950° N, 123.70634° W. Detrital zircon sample 16 was collected from it. The Y-axis indicates stratigraphic height above the base of section (0 m; bottom left) where the base of section represents the oldest strata of the Nanaimo Group exposed at the Bare Point outcrop location. Paleoenvironment interpretations and additional information can be found in Jones, et al.^37^.


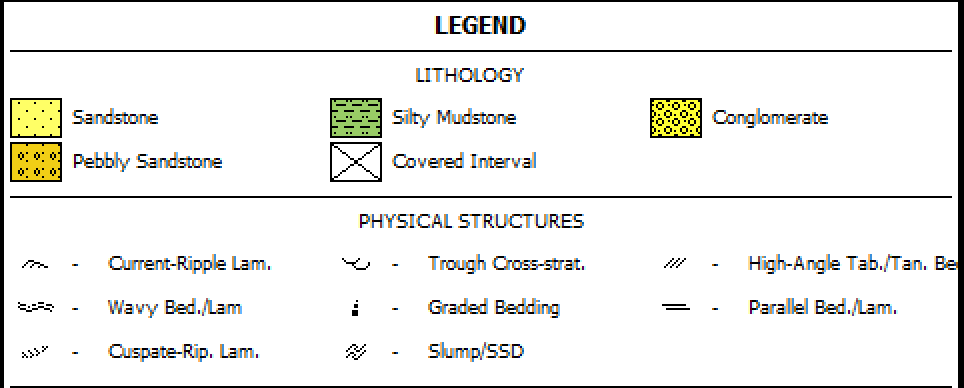


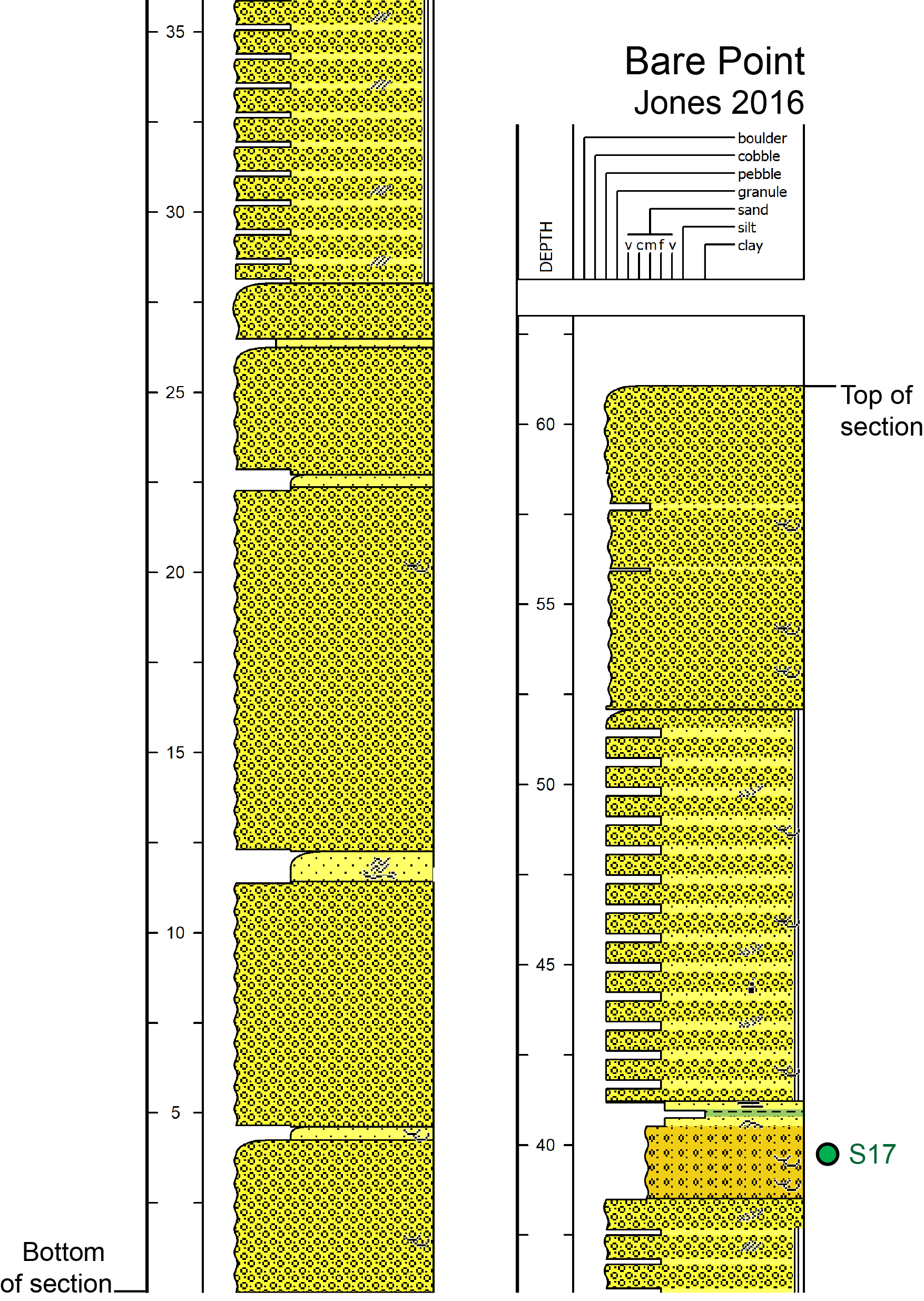


**Trestle 66**

The Trestle 66 outcrop is located in Cowichan River Provincial Park, Vancouver Island. The base of section is at 48.77588° N, 123.92860° W. Detrital zircon sample 18 was collected from it. The Y-axis indicates stratigraphic height above the base of section, (0 m; bottom left) where the base of section represents the oldest logged strata of the Nanaimo Group exposed at the Trestle 66 outcrop location. Paleoenvironment interpretations and additional information can be found in Jones ^37^.


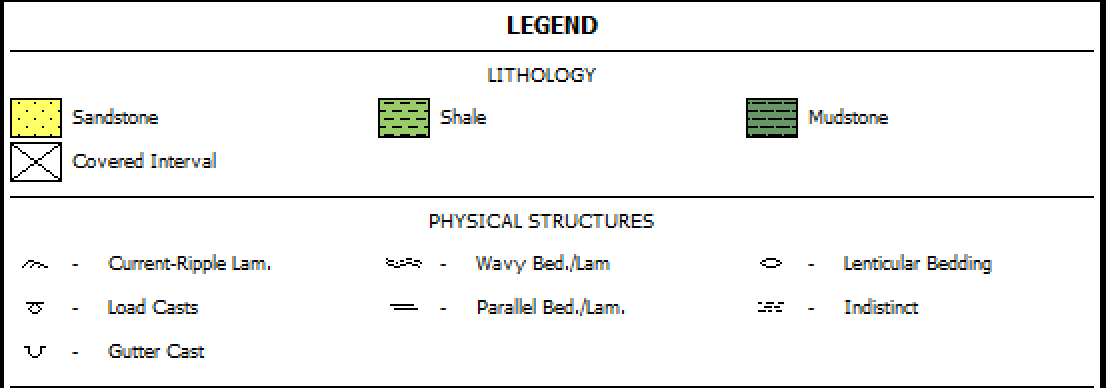


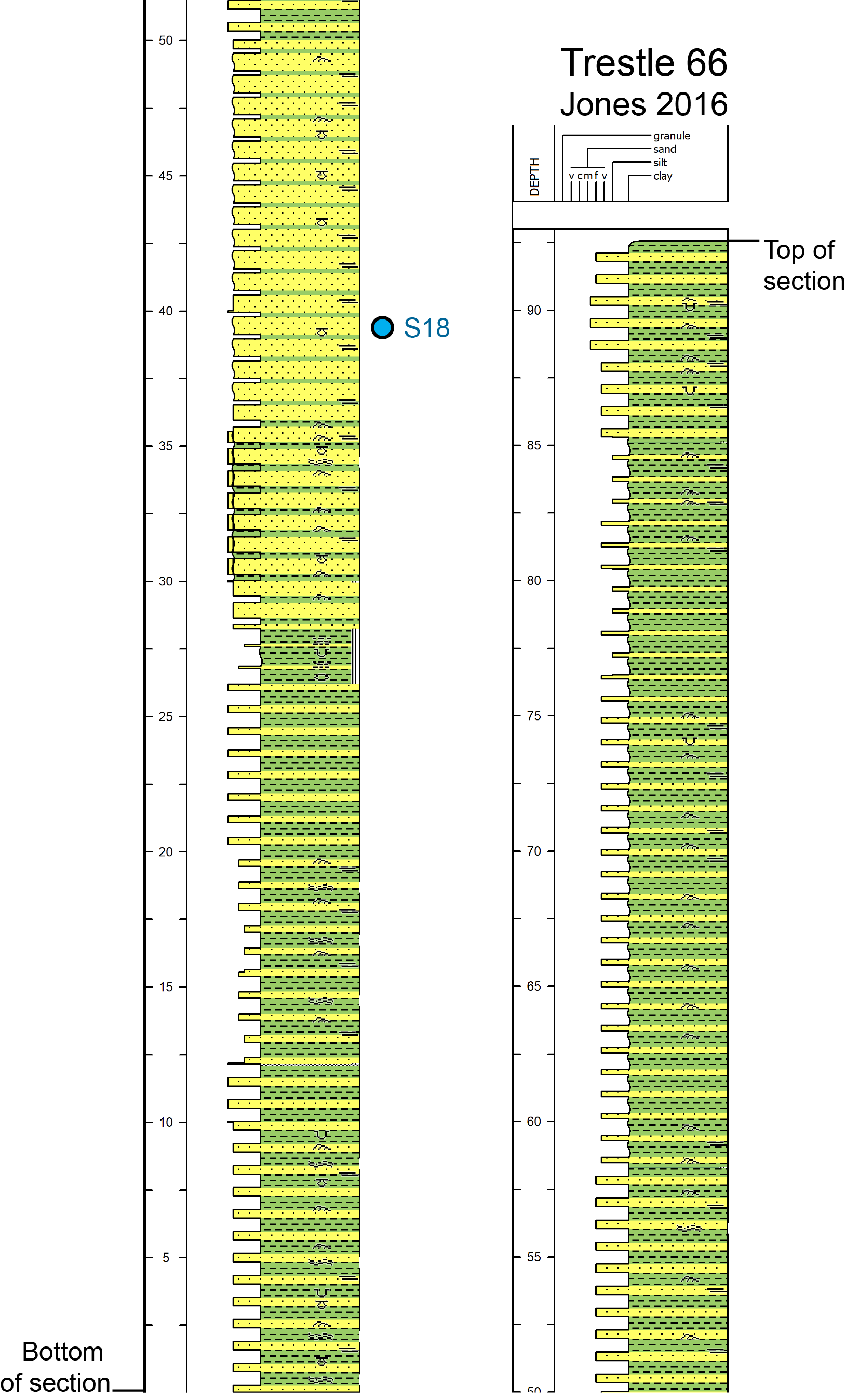


**Ruckle Park**

The Ruckle Park outcrop is located in Ruckle Park, Salt Spring Island. The base of section is at 48.76665° N, 123.37236° W. Detrital zircon samples 20 and 21 were collected from it. The Y-axis indicates stratigraphic height above the base of section (approximately 1 m; bottom left) where the base of section represents the oldest strata of the Nanaimo Group. Paleoenvironment interpretations and additional information can be found in Jones ^38^.


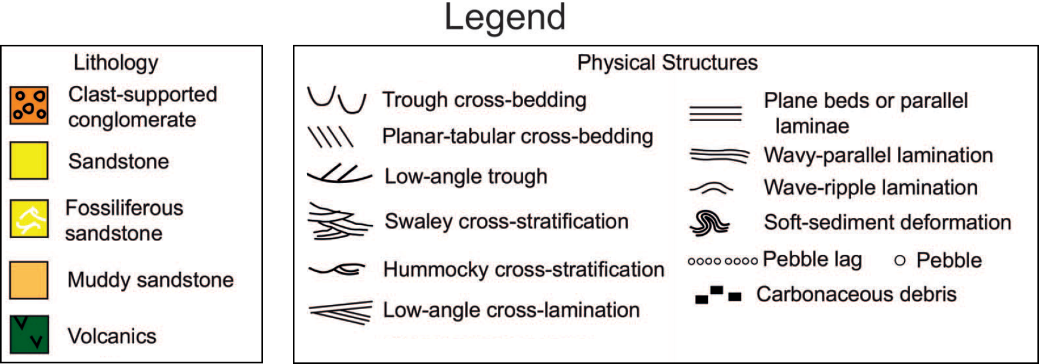


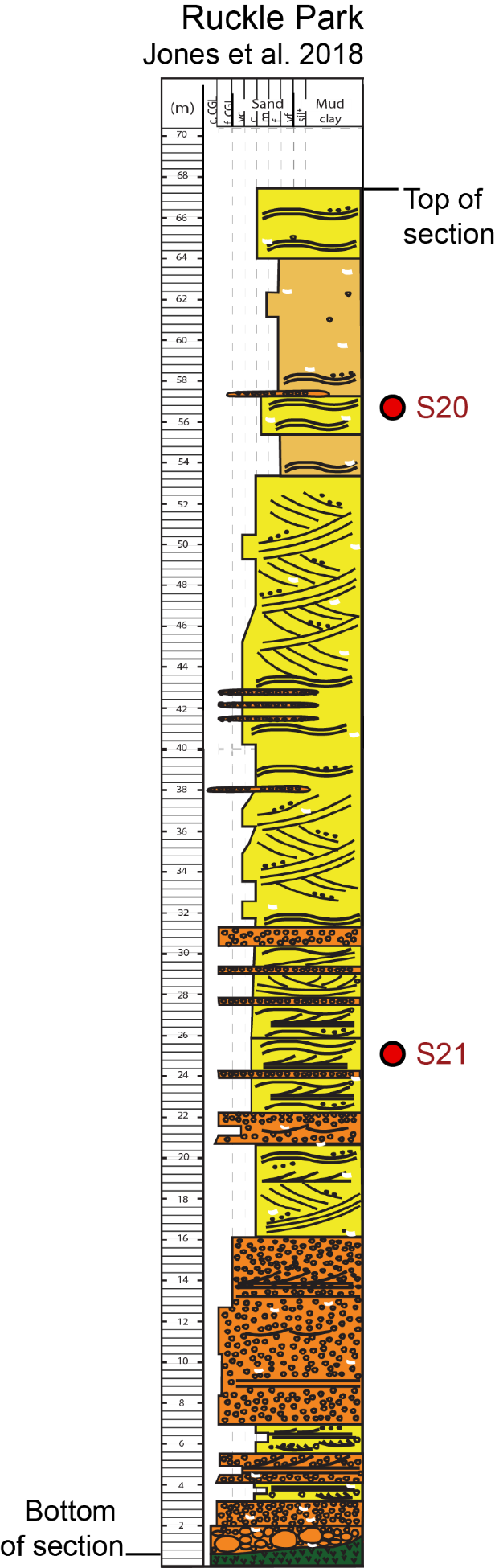

Supplement: Supplementary file 2 — Supplementary Dataset 2 [file 41598_2019_51795_MOESM2_ESM.docx]
